# Current landscape and future directions of bispecific antibodies in cancer immunotherapy

**DOI:** 10.3389/fimmu.2022.1035276

**Published:** 2022-10-28

**Authors:** Jing Wei, Yueyao Yang, Gang Wang, Ming Liu

**Affiliations:** ^1^ Gastric Cancer Center/Cancer Center, West China Hospital, Sichuan University, Chengdu, China; ^2^ National Engineering Research Center for Biomaterials, Sichuan University, Chengdu, China

**Keywords:** bispecific antibodies, bispecific T cell engager, cancer immunotherapy, tumor microenvironment (TME), clinical trials

## Abstract

Recent advances in cancer immunotherapy using monoclonal antibodies have dramatically revolutionized the therapeutic strategy against advanced malignancies, inspiring the exploration of various types of therapeutic antibodies. Bispecific antibodies (BsAbs) are recombinant molecules containing two different antigens or epitopes identifying binding domains. Bispecific antibody-based tumor immunotherapy has gained broad potential in preclinical and clinical investigations in a variety of tumor types following regulatory approval of newly developed technologies involving bispecific and multispecific antibodies. Meanwhile, a series of challenges such as antibody immunogenicity, tumor heterogeneity, low response rate, treatment resistance, and systemic adverse effects hinder the application of BsAbs. In this review, we provide insights into the various architecture of BsAbs, focus on BsAbs’ alternative different mechanisms of action and clinical progression, and discuss relevant approaches to overcome existing challenges in BsAbs clinical application.

## 1 Introduction

**Figure 1:**
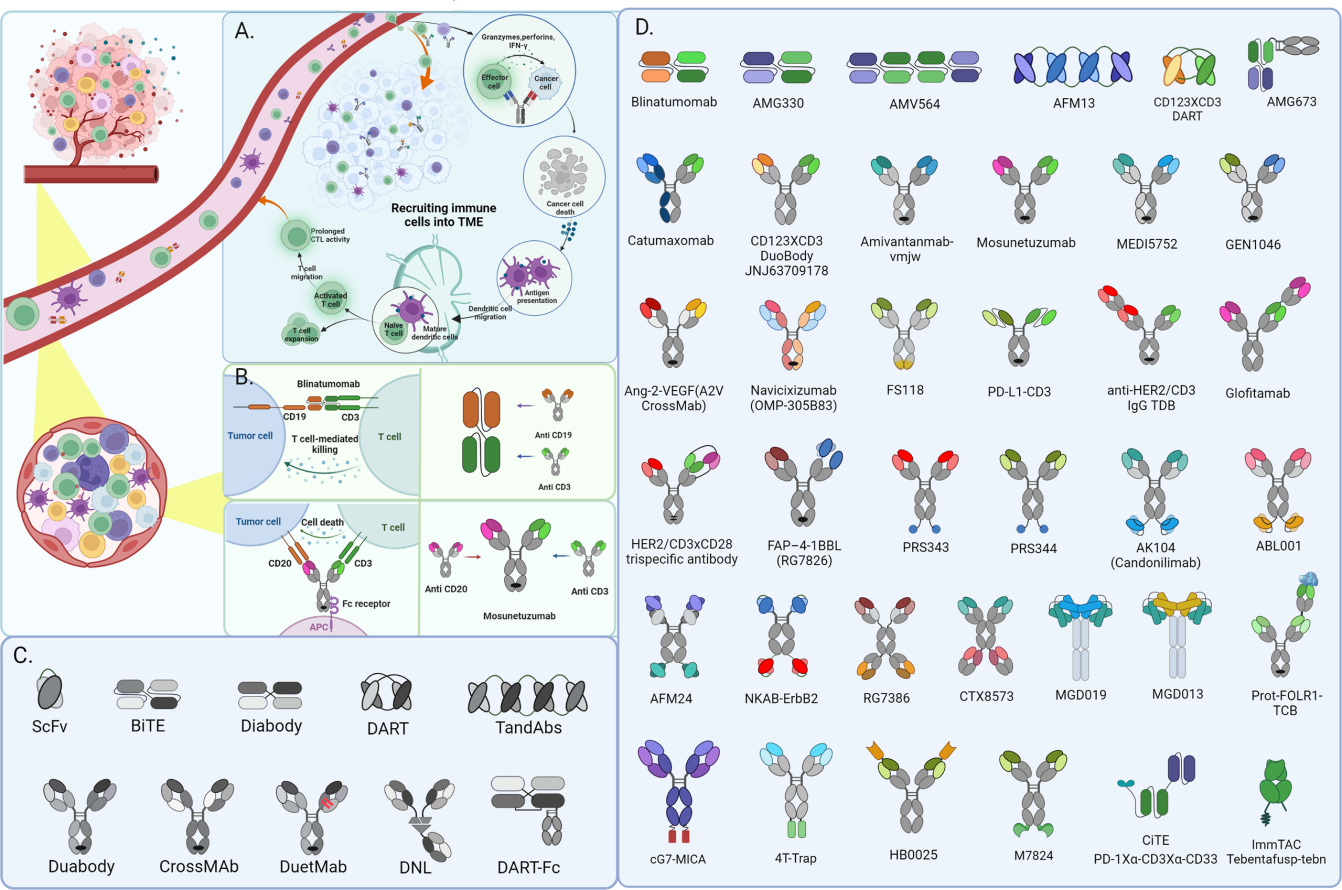
CD3+ bispecific T-cell engaging antibodies exert function in Hematological Malignancies and recruit immune cells into the solid tumor microenvironment for cancer immunotherapy. The schematic depicts the mechanism of action of BsAbs in solid tumors **(A)** and Hematological Malignancies **(B)**. besides, there shows partial fragments of antibody as well as the derivatives formats constructed from them in diagram **(C)** and various architecture of BsAbs in diagram **(D)** mentioned in this review.

Immunotherapy breakthroughs in cancer treatment with synthetic multifunctional biotherapeutics have fueled cancer immunotherapy and the exploration of antibody alternative modes of action (1, 2). Conventional targeted or immunotherapeutic drugs can only be used to inhibit one or one class of targets, thus giving birth to some unique combination drug regimens. Currently, Constant engineering technical breakthroughs in antibody development have aided in producing many BsAb designs (3) (**Figures 1C, D**). Bispecific antibodies, constructed *via* quadroma, chemical conjugation, and genetic recombination (4), exert effector functions beyond natural antibodies through redirecting cells or modulating different pathways, providing numerous possibilities for therapeutic application and contributing to improving treatment responses in refractory tumor patients. Although more than a hundred BsAbs are currently under clinical evaluation in cancer treatment, most are still in the early stages (4), and only four BsAbs have been approved by FDA (**Table 1**). These include Catumaxomab (Fresenius/Trion’s Removab^®^) which was withdrawn from the market in 2017, Blinatumomab (Amgen’s Blincyto^®^), Amivantamab-vmjw (Janssen’s Rybrevant^®^), and Tebentafusp-tebn (Immunocore’s Kimmtrak^®^) (5–11).

**Table 1:** Bispecific Antibody Approved by the FDA.

Antibody/Interventions	Target	Platform/Format	Company	Conditions	Year of Approval	Status
Catumaxomab (removab)	CD3 × EpCAM	TrioMab	TriOn Pharma Ltd.	EpCAM positive ovarian and gastric cancer	2009	Withdrawn from the market in 2017
Blinatumomab (Blincyto; MT103)	CD3 × CD19	BiTE^®^	Amgen	Hematological	2014	marketed
Amivantamab (JNJ-61186372)	EGFR × MET	Duobody	Johnson & Johnson	Non-small cell lung cancer	2021	marketed
Tebentafusp (tebentafusp-tebn; Kimmtrak^®^)	CD3 × IMCgp100	ImmTAC^®^	Immunocore Ltd. & AstraZeneca	Uveal melanoma	2022	marketed

Furthermore, multiple studies investigate the mechanisms of action by which BsAbs detect various tumor targets such as angiogenesis, proliferation, invasion, and immune modulation. However, potential immunotherapy side effects must be considered, whereas toxicity in normal tissues and systemic immune responses limit the use of BsAbs (12–15). We concentrate on the advances in BsAbs design, mechanisms of action, and clinical trial development in this review (**Table 2**). We also talk about difficulties and potential solutions for enhancing drug delivery.

**Table 2:** Bispecific Antibody Clinical Trials Ongoing.

Target	Antibody/Interventions	Platform/Format	Company	Conditions	Phases	NCT Number	Status	Start Date	Completion Date
CD3 × CD19	Blinatumomab	BiTE^®^	Amgen	B-precursor ALL;B-Cell ALL;DLBCL;Adult PH-Positive ALL	Phase 4	NCT04506086	Recruiting	2021-08-26	2025-12-28
					Phase 1	NCT03751709	Recruiting	2020-02-14	2024-02-01
					Phase 3	NCT03643276	Recruiting	2018-07-15	2028-07-14
					Phase 3	NCT04722848	Recruiting	2021-09-08	2027-09-01
CD3 × CD19	TNB-486	UniAb	AstraZeneca(TeneoOne Inc)	B-cell NHL	Phase 1	NCT04594642	Recruiting	2021-03-02	2024-05-01
CD3 × CD20	Odronextamab	DouBody	Regeneron	B-cell NHL	Phase 2	NCT03888105	Recruiting	2019-11-13	2028-02-04
CD3 × CD20	Odronextamab	Veloci-Bi	Regeneron	NHL	Phase 1	NCT02290951	Recruiting	2015-01-09	2025-12-02
CD3 × CD20	glofitamab(RO7082859)	“2:1” TCB BsAb	Roche	RR Lymphomas	Phase 2	NCT04703686	Recruiting	2021-03-30	2025-03-01
CD3 × CD20	GB261	TEA	Genor BioPharma	B Cell NHL;CLL	Phase 1/2	NCT04923048	Recruiting	2021-08-31	2025-06-28
CD3 × CD22	JNJ-75348780	UniAb ^®^	Johnson & Johnson	NHL;CLL;Lymphoma	Phase 1	NCT04540796	Recruiting	2020-11-20	2024-03-05
CD3 × CD38	ISB 1342	BEAT^®^	Ichnos Sciences	RR Multiple Myeloma	Phase 1	NCT03309111	Recruiting	2017-10-25	2024-05-01
CD3 × CD38	Y150	YBODY^®^	Wuhan YZY Biopharma Co.,Ltd.	RR Multiple Myeloma	Phase 1	NCT05011097	Recruiting	2021-07-08	2024-12-30
CD3 × DLL3	Tarlatamab(AMG 757)	HLE BiTE^®^	Amgen	Neuroendocrine Prostate Cancer;Extensive Stage SCLC	Phase 1	NCT04702737	Recruiting	2021-06-10	2026-04-10
					Phase 1	NCT03319940	Recruiting	2017-12-26	2025-02-20
CD3 × EGFR	MVC-101 (TAK-186)	COBRATM	Maverick Therapeutics	Advanced Cancer	Phase 1/2	NCT04844073	Recruiting	2021-03-08	2024-10-01
CD3 × FLT3	CLN-049	TCE IgG format	Cullinan Oncology	RR AML	Phase 1	NCT05143996	Recruiting	2021-11-18	2022-07-22
CD3 × STEAP1	AMG 509	XmAb ^®^	Amgen	Prostate Cancer	Phase 1	NCT04221542	Recruiting	2020-03-04	2027-06-07
CD3 × MUC16	REGN4018	BiTE^®^	Regeneron	Recurrent Ovarian Cancer	Phase 1/2	NCT03564340	Recruiting	2018-05-21	2024-07-24
CD3 × MUC17	AMG 199	HLE BiTE^®^	Amgen	MUC17-positive Solid Tumors	Phase 1	NCT04117958	Recruiting	2020-01-20	2025-04-01
CD3 × PD-1	ONO-4685	Biclonics ^®^	Ono-pharma	RR T Cell Lymphoma	Phase 1	NCT05079282	Recruiting	2021-12-10	2026-06-01
CD3 × GPRC5D	Talquetamab (JNJ-64407564)	DuoBody ^®^	Johnson & Johnson	Hematological Malignancies; RR Multiple Myeloma	Phase 1	NCT04773522	Recruiting	2021-05-20	2023-01-09
					Phase 1	NCT03399799	Recruiting	2017-12-16	2025-03-03
					Phase 2	NCT04634552	Recruiting	2021-02-01	2026-04-13
					Phase 1	NCT04586426	Recruiting	2020-12-15	2024-05-28
CD3 × BCMA	Teclistamab	DuoBody ^®^	Johnson & Johnson	Hematological Malignancies; RR Multiple Myeloma	Phase 1	NCT05338775	Recruiting	2022-05-25	2025-10-15
					Phase 1	NCT03145181	Recruiting	2017-05-16	2025-01-23
					Phase 1/2	NCT04696809	Recruiting	2021-02-22	2025-06-30
					Phase 2	NCT04557098	Recruiting	2020-09-17	2024-12-30
					Phase 1	NCT04586426	Recruiting	2020-12-15	2024-05-28
CD3 × BCMA	TNB-383B	UniAb^®^	Abbvie	Multiple Myeloma	Phase 1/2	NCT03933735	Recruiting	2019-06-24	2025-08-24
CD3 × BCMA	REGN5458	VelocImmune ^®^	Regeneron	Multiple Myeloma	Phase 1/2	NCT03761108	Recruiting	2019-01-23	2029-10-11
CD3 × BCMA	Elranatamab (PF-06863135)	DuoBody	Pfizer Inc.	RR Multiple Myeloma; Multiple Myeloma	Phase 2	NCT05228470	Recruiting	2021-12-21	2025-02-05
					Phase 3	NCT05020236	Recruiting	2021-10-04	2026-02-24
CD3 × BCMA	EMB-06	FIT-Ig^®^	EpimAb Biotherapeutics	RR Multiple Myeloma	Phase 1/2	NCT04735575	Recruiting	2021-05-20	2025-03-01
CD3 × PSMA	AMG 340	BiTE^®^	Amgen	Metastatic Castration-resistant Prostate Cancer	Phase 1	NCT04740034	Recruiting	2021-04-29	2024-09-30
CD3 × PSMA	CC-1		University Hospital Tuebingen	LCSC	Phase 1/2	NCT04496674	Recruiting	2022-02-02	2025-09-30
				Castration-Resistant Prostatic Cancer	Phase 1	NCT04104607	Recruiting	2019-11-15	2022-09-01
CD3 × PSCA	GEM3PSCA	TCE	GEMoaB	NSCLC	Phase 1	NCT03927573	Recruiting	2019-04-15	2023-06-01
PD-1 × CTLA-4	Cadonilimab (AK104)	Tetrabody	Akeso, Inc.	Advanced Malignant Tumors	Phase 1/2	NCT05235542	Recruiting	2022-07-12	2024-03-01
					Phase 2	NCT04728321	Recruiting	2021-01-27	2023-03-31
					Phase 1	NCT04572152	Recruiting	2021-01-18	2023-12-01
PD-1 × CTLA-4	SI-B003		Sichuan Baili Pharmaceutical Co.	Advanced Solid Tumors	Phase 1	NCT04606472	Recruiting	2020-11-19	2022-09-22
PD-1 × CTLA-4	MEDI5752	DuetMab	AstraZeneca	Selected Advanced Solid Tumors	Phase 1	NCT03530397	Recruiting	2018-04-24	2024-03-20
PD-1 × VEGF	AK112	TETRABODY	Akeso, Inc.	Advanced Malignant Tumors	Phase 2	NCT04870177	Recruiting	2021-04-09	2024-05-01
					Phase 1/2	NCT05229497	Recruiting	2022-05-04	2024-02-01
					Phase 1/2	NCT05214482	Recruiting	2022-01-25	2024-01-25
					Phase 1	NCT05116007	Recruiting	2021-03-29	2022-12-22
					Phase 1/2	NCT04999605	Recruiting	2021-07-22	2024-06-01
					Phase 1/2	NCT04900363	Recruiting	2021-05-14	2024-05-01
					Phase 1	NCT04047290	Recruiting	2019-09-20	2024-02-28
					Phase 2	NCT04736823	Recruiting	2021-02-01	2024-01-01
PD-1 × PD-L1	IBI318	SEBA	Innoventbio.	Non-Small Cell Lung Cancer	Phase 1	NCT04777084	Recruiting	2021-08-01	2023-05-01
PD-1 × LAG-3	EMB-02	FIT-Ig^®^	EpimAb Biotherapeutics	Advanced Solid Tumors	Phase 1/2	NCT04618393	Recruiting	2021-03-11	2025-12-31
PD-1 × LAG3	RO7247669		Roche	Solid Tumors;Esophageal Squamous Cell Carcinoma	Phase 1	NCT04140500	Recruiting	2019-11-11	2025-12-31
					Phase 2	NCT04785820	Recruiting	2021-06-25	2025-05-31
PD-1 × TIM3	AZD7789		AstraZeneca	Relapsed/Refractory Classical HL;NSCLC	Phase 1/2	NCT05216835	Recruiting	2022-03-18	2025-09-26
					Phase 1/2	NCT04931654	Recruiting	2021-09-28	2025-07-01
PD-1 × TIM-3	RO7121661	CrossMab	Roche	Solid Tumors;Esophageal Squamous Cell Carcinoma	Phase 2	NCT04785820	Recruiting	2021-06-25	2025-05-31
PD-1 × TIGIT	AZD2936		AstraZeneca	NSCLC	Phase 1/2	NCT04995523	Recruiting	2021-09-14	2025-07-14
PD-1 × HER2	IBI315	PentamBODY™	Innoventbio.	Advanced Solid Tumor	Phase 1	NCT04162327	Recruiting	2019-11-26	2025-12-30
PD-L1 × CTLA4	KN046	sdAb	Alphamab Oncology	Solid Tumors;Thymic Carcinoma; Advanced Renal Cell Carcinoma	Phase 2	NCT04469725	Recruiting	2020-12-02	2023-08-31
					Phase 1	NCT04522323	Recruiting	2020-08-05	2024-02-09
PD-L1 × TIGIT	HLX301	Nanobody	Henlius	Solid Tumors	Phase 1/2	NCT05390528	Recruiting	2022-06-20	2024-12-30
					Phase 1/2	NCT05102214	Recruiting	2022-05-03	2022-02-24
PD-L1 × LAG-3	ABL501	Grabody™ I platform	Ablbio.	Solid Tumors	Phase 1	NCT05101109	Recruiting	2021-10-06	2023-07-31
PD-L1 × LAG-3	FS118	mAb2™	F-Star Therapeutics Inc.	Advanced Cancer	Phase 1/2	NCT03440437	Recruiting	2018-04-16	2022-12-22
PD-L1 × 4-1BB(CD137)	PRS-344/S095012	Anticalin^®^ technology platform	Pieris Pharmaceuticals, Inc.	Solid Tumor	Phase 1/2	NCT05159388	Recruiting	2021-09-08	2024-11-15
PD-L1 × 4-1BB(CD137)	ABL503	Grabody-T	I-mabbiopharma. & ABL Bio	Advanced Solid Tumor	Phase 1	NCT04762641	Recruiting	2021-04-01	2023-06-15
PD-L1 × 4-1BB(CD137)	FS222	tetravalent BsAb	F-Star Therapeutics Inc.	Advanced Cancer	Phase 1	NCT04740424	Recruiting	2020-12-14	2025-05-01
PD-L1 × 4-1BB(CD137)	MCLA-145	Biclonics ^®^	Merus	Advanced Solid Tumor	Phase 1	NCT03922204	Recruiting	2019-05-08	2022-12-22
PD-L1 × CD27	CDX-527	IgG1-ScFv	Celldex Therapeutics	Advanced Malignancies	Phase 1	NCT04440943	Recruiting	2020-08-04	2024-03-01
PD-L1 × TGF-β	Y101D	CHECKBODY™	Wuhan YZY Biopharma Co.,Ltd.	Advanced Solid Tumors	Phase 1	NCT05028556	Recruiting	2021-08-06	2023-08-06
PD-L1 × TGF-β	QLS31901	Tetravalent BsAb	Qilu-pharma.	Advanced Malignant Tumor	Phase 1	NCT04954456	Recruiting	2021-06-02	2022-12-30
OX40 × CD137	FS120	mAb2™	F-Star Therapeutics Inc.	Advanced Cancer	Phase 1	NCT04648202	Recruiting	2020-11-18	2023-11-01
EGFR × CD28	REGN7075	human Ig4 bsAb	Regeneron Pharmaceuticals	Advanced Solid Tumors	Phase 1/2	NCT04626635	Recruiting	2020-12-21	2025-04-03
EGFR × 4-1BB	HLX35		Henlius	Solid Tumors	Phase 1	NCT05360381	Recruiting	2022-06-03	2024-12-30
EGFR × c-Met	MCLA-129	Biclonics ^®^	Bettapharma. & Merus	Advanced NSCLC and Solid Tumors	Phase 1/2	NCT04930432	Recruiting	2021-09-24	2025-06-30
					Phase 1/2	NCT04868877	Recruiting	2021-04-28	2025-04-01
EGFR × c-Met	EMB-01	FIT Ig^®^	EpimAb Biotherapeutics	Metastatic NSCLC;Neoplasms	Phase 1/2	NCT03797391	Recruiting	2018-12-13	2023-03-14
					Phase 1/2	NCT05176665	Recruiting	2021-10-21	2024-08-31
EGFR × cMet	Amivantamab	Genmab’s DuoBody^®^	Johnson & Johnson	Advanced Solid Malignancies;Non-Small-Cell Lung Cancer	Phase 1	NCT02609776	Recruiting	2016-05-24	2024-01-26
					Phase 1	NCT04606381	Recruiting	2020-11-10	2024-10-02
					Phase 1	NCT04077463	Recruiting	2019-09-04	2024-12-30
HER2 × HER3	zenocutuzumab (MCLA-128)	Biclonics^®^	Merus N.V.	Breast Cancer Metastatic;Solid Tumors Harboring NRG1 Fusion	Phase 2	NCT02912949	Recruiting	2022-01-15	2024-12-31
HER2(ECD2) × HER2(ECD4)	ZW25 (Zanidatamab)	Azymetric™	Beigene.	HER2-expressing Cancers	Phase 3	NCT05152147	Recruiting	2021-12-02	2025-07-01
					Phase 2	NCT04224272	Recruiting	2020-06-10	2023-06-30
					Phase 2	NCT03929666	Recruiting	2019-08-29	2024-04-30
HER2(ECD2) × HER2(ECD4)	MBS301	glyco-engineered IgG1 BsAb	Mab-works.	HER2-positive Solid Tumor	Phase 1	NCT03842085	Recruiting	2019-04-11	2022-12-22
HER2 × 4-1BB	Cinrebafusp alfa (PRS-343)	Anticalin^®^ technology platform	Pieris Pharmaceuticals, Inc.	HER2-positive Solid Tumor	Phase 2	NCT05190445	Recruiting	2021-11-01	2023-02-01
HER2 × CD47	IMM2902	mAb-Trap	Immuneonco.	HER2-expressing Advanced Solid Tumors	Phase 1	NCT05076591	Recruiting	2022-06-20	2024-12-01
IMCgp100 × CD3	Tebentafusp (IMCgp100)	ImmTAC ^®^	Immunocore Ltd. & AstraZeneca	Advanced Uveal Melanoma;Malignant Melanoma	Phase 1/2	NCT02535078	Recruiting	2022-11-15	2025-01-01
CD3 × PRAME	IMC-F106C	ImmTAC ^®^	Immunocore Ltd	Select Advanced Solid Tumors	Phase 1/2	NCT04262466	Recruiting	2020-02-25	2026-02-01
CD47 × MSLN	NI-1801		Light Chain Bioscience - Novimmune SA	Mesothelin Expressing Solid Cancers	Phase 1	NCT05403554	Recruiting	2022-04-29	2025-09-30
CD47 × CD19	TG-1801		TG Therapeutics, Inc.	B-Cell Lymphoma	Phase 1	NCT03804996	Recruiting	2019-03-05	2022-12-22
PSMA ×	LAVA-1207	Gammabody™	lavatherapeutics.	Castration Resistant Prostate Cancer	Phase 1/2	NCT05369000	Recruiting	2022-06-27	2024-03-01
MUC16 × CD28	REGN5668/REGN4018	BiTE^®^	Regeneron Pharmaceuticals	Ovarian Cancer;Fallopian Tube Cancer;Primary Peritoneal Cancer	Phase 1/2	NCT04590326	Recruiting	2020-12-09	2026-07-28
CD30 × CD16A	AFM13	ICE^®^	Affimed	Lymphoma	Phase 1/2	NCT04074746	Recruiting	2020-07-18	2025-04-15
MAGEA4 × MAGEA8	IMA401	TCER ^®^	Immatics Biotechnologies GmbH & Bristol-Myers Squibb	Refractory/Recurrent Cancer	Phase 1	NCT05359445	Recruiting	2022-05-19	2027-11-01
DLL4 × VEGF-A	CTX-009 (ABL001)		Handok Inc., Compass Therapeutics & ABL Bio, Inc.	Advanced Solid Tumors;Biliary Tract Cancer	Phase 1/2	NCT04492033	Recruiting	2020-06-22	2025-07-31

RR ALL, Relapsed or Refractory Acute Lymphocytic Leukemia; DLBCL, diffuse large B-cell lymphoma; NHL, Non Hodgkin Lymphoma; CLL, Chronic Lymphocytic Leukemia; NSCLC, Non Small Cell Lung Cancer; LCSC, Lung Cancer Squamous Cell; SCLC, Small Cell Lung Cancer.

## 2 BsAb construct formats

In natural bivalent antibodies, the two antigen binding sites are identical and consist of variable regions of the heavy chain and light chain. Bispecific antibodies (BsAbs) are dual-specificity molecules binding two different epitopes simultaneously, the concept of which has been first described decades ago by Nisonoff et al. (1). Since there are no naturally occurring bispecific antibodies, BsAbs were initially developed by chemically coupling two monoclonal antibody fragments or creating quadroma cell lines combined with two homologous hybridomas. The field of recombinant bispecific antibodies for diagnostic and therapeutic purposes has been transformed by the quickly developing engineering technologies and pharmaceutical industry, leading to a variety of BsAbs with varying size, half-life, valency, flexibility, and permeability (2). Recombinant DNA technology is now the most used technique for producing bispecific antibodies.

IgG-like antibody types (containing an Fc unit) and non-IgG-like (without an Fc unit) antibody formats are the two broad categories into which BsAbs can be generally categorized (3). The intention of this classification mechanism well emphasizes the existence of the Fc domain, which not only facilitates the functionality mentioned above but contributes to the solubility, stability, and purification of the BsAbs (4). Additionally, this region can be genetically altered to abolish antibody-dependent cell-mediated cytotoxicity (ADCC) or complement dependent cytotoxicity (CDC) while retaining the potential for a lengthy half-life (5). Although non-IgG-like antibody formats exert therapeutic activities depending on antigen-recognition domains, smaller size enables them to enhance tissue penetration while rapid renal clearance results in a relatively short plasma half-life.

Heavy and light chain mispairing poses serious problems for bispecific antibodies made in IgG formats from two distinct polypeptides, leading to ineffective antibodies or unwanted homodimers. In the interim, mitigation measures have been taken for such issues. For example, the “knobs-into-hole” approach has been developed to mutate the corresponding amino acid size of the third constant domain of the antibody for correctly pairing the heavy chain (6). In contrast, “the light chain mispairing” problems can be well circumvented by the “CrossMab” strategy by swapping the CL (light chain constant region) domain of the light chain with the corresponding CH1 domain of the heavy chain to construct a correct light chain association (7, 12). Other established and mature techniques to prevent light chain mispairing include Eli Lilly’s “Orthogonal Fab interface” by introducing amino acid mutations to the light chain and the tcBsIgG (tethered-variable CL bispecific IgG) platform developed by the Genentech, which focuses on linking the VL (light chain variable region) to VH (heavy chain variable region) *via* the G_4_S linker (8, 9). In addition, the asymmetric heavy chains also form the basis for constructing multi-specific antibodies, such as trispecific, trivalent, or tetravalent antibodies, with greater targeting specificities for cancer therapy.

The production of non-IgG-like BsAbs without an Fc unit can be accomplished using Fab fragments or by joining the variable light-heavy domains of two antibodies. These antibodies can be broadly categorized into scFv (single-chain variable fragment)-based bsAbs, Nanobodies, Dock-and-lock (DNL) method-building antibodies, and other bispecific/multi-specific molecules (10). The specificity and ability to bind to antigens of full-length antibodies are maintained by scFv, composed of the VL and VH domains connected by a flexible peptide. Additionally, the linker length significantly impacts how scFv molecules associate, resulting in various polymerizations such as dimers, trimers, or tetramers. The Tandem scFvs constructed by the Bispecific T-cell Engager (BiTE^®^) antibody platform link two scFvs with a repeat glycine-serine short motifs (11), which enables antigen recognizing sites to rotate flexibly.

In contrast, the linking method of the Diabody format is slightly different from Tandem scFvs (13). Heterodimerization of those fragments is induced by crossing the VH and VL domains of the two scFvs by two shorter polypeptide chains. Even if the two peptides linker facilitates the molecular stability to a certain extent, the intramolecular association between the VH and VL domains from the same scFv is hampered (14).

Further research on crystal structures suggests that the Fv contact’s instability impacts overall flexibility, and the diabodies are too rigid to crystallize. As a result, “Dual-affinity retargeting molecules”—an inter-chain disulfide bond—have been created to strengthen the structural stability of the diabodies (DARTs) (15, 16). However, the small size of this format makes the DARTs molecule rapidly eliminate from the serum, and this issue has been well solved by the strategy of Fc fragment fusion to the part of the DARTs, promoting FcRn-mediated recycling at the same time. The emergence of this constructive format of bispecific antibodies called “DART-Fc” was designed by MacroGenics. Besides, the polymerization of two diabodies connected by two polypeptide chains forms the “Tandem diabodies” (TandAbs), the tetravalent derivatives possessing two antigen-recognizing moieties for each antigen.

In addition to being synthesized from the constituents of various antibodies, BsAbs can be fused to other protein domains to improve their functions for future adaptive therapeutic uses. For example, the scFv-based BsAbs have relatively great tissue permeability and decreased immunogenicity for the lack of Fc unit. However, short half-lives caused by relatively low molecular mass will affect the serum circulating level, which induces the Increased administration and doses of therapeutic agents, thus limiting the clinical promoting application. The format “scFv-HSA-scFv”, fusing two scFvs to the albumin, and conjugation of Polyethylene glycol PEGylation are available application strategies for circulating half-life extension (17). Furthermore, the Dock-and-lock (DNL) method, creating multivalent and multifunctional antibody derivatives such as trivalent bispecific antibodies by heterodimerizing protein domains fused with Fab domains or integral antibodies, is another available antibody platform enabling more promising antibody construction with retained bioactivity (18) (**Figure 1C**).

## 3 BsAbs redirecting immune effector cells and reactivating anti-tumor immunity

### 3.1 Bispecific T-Cell Engagers (BiTE) recruiting adaptive effector cells for tumor redirection

Adaptive immunity is essential for monitoring and suppressing tumorigenesis and cancerous progression. T-cells are the focal point of many immunotherapies and have a potent tumor-killing effect as a crucial part of adaptive immunity. BiTE is a potential immunological drug that directs T lymphocytes against tumor cells to treat a variety of malignancies. The ideal BiTE molecular design needs to consider the following factors: firstly, a suitable CD3 binding arm should be selected; secondly, the affinity between TAA binding sites and anti-CD3 units should differ by at least 10-fold to achieve sequential binding to tumor cells and immune cells, thus reducing the risk of severe cytokine release syndrome (CRS); finally, an appropriate physical distance between the two antibody binding units can also play a role in improving the efficacy and reducing the risk of CRS.

The high affinity of CD3 binding will allow BiTE to occupy CD3 antigen and activate T cells continuously, eventually leading to T cell depletion. However, suitable affinity will bind to and dissociate from the CD3 antigen of distinct T cells and the repeated binding to T cells will cause a “waterfall effect” to cluster T cells, ultimately resulting in many T cells activating to attain the greatest tumor-killing effect. In addition, high CD3 binding affinity will make BiTE more concentrated in the spleen, thus making it difficult to reach tumor tissues, especially solid tumors. Physically bridging T cells with tumor cells by BiTEs enables catalyzing the formation of an optimal immunologic synapse, which’s important for T cells activation and robust cytotoxicity to target tumor cells, ultimately leading to apoptosis *via* membrane disruption mediated by perforin releasing. In addition, multiple cytokine secretion from T cells activated by BiTEs, like IL-2, IFN-γ, and TNF-α, enhance their effectiveness in the anti-tumor function (**Figures 1A, B**). Strategies that harness the potential of T cells to identify and kill cancer cells in a targeted manner have ushered in a new era of cancer therapy and led to the development of a wide range of immunotherapy devices.

#### 3.1.1 BiTEs in hematologic malignancies

Due to the distinct traits of hematopoietic malignancies, immunotherapy for leukemia and lymphoma has assumed a leadership role and made significant advancements. Given the unique property of the hematological system, malignancy cells constantly interact with immune cells, making it easier for BiTE to exert anti-tumor actions (19). Among various forms of immunotherapy, engaging T cells in hematological malignancies mediated by bispecific antibodies (BsAbs) has been demonstrated as an attractive strategy for providing alternative treatment options for recurrent and/or refractory hematological malignancies patients (20). Bispecific T-cell engagers (BiTEs), consisting of two binding sites simultaneously for a selective tumor antigen and CD3 molecule expressed on host T cells, has been emerged as the most promising BsAb form (**Figure 1B**). A diverse variety of BiTEs have emerged for cancer immunotherapy, and the specific targets are mainly CD19, CD20, CD123, CD33, CD38, and B-cell maturation antigen (BCMA) in hematological malignancies. Ideal target antigens must satisfy the criteria uniquely expressed on malignant cells to avoid on-target/off-cancer toxicity and reduce the possibility of antigen-loss variants. Even though few target antigens satisfy the above demands simultaneously (21, 22), various types of BiTE with different antigen-recognition domains have been under exploration for hematological malignancies.

Blinatumomab (MT103), the first BiTE tested in clinical trials specifically designed to target T cells based on the recognition of CD3ϵ to CD19 expressing B cell hematologic malignancies, can induce immune-mediated B-cell lymphoblasts lysis led by cytotoxic T cells. CD19 is a transmembrane molecule relatively specific to B cells persisting throughout B-cell differentiation and existed on the surface of most B cell hematologic malignancies, which is a superior target for cancer immunotherapy achieving exceptional curative effect with R/R B cell ALL (Acute Lymphocytic Leukemia) patients. And the body’s function will not be seriously affected while the missing of normal B cells or bone marrow cells can be continuously replenished by hematopoietic stem cells.

A 7.1-month overall survival and a CR/CRh of 36%, including partial patients with a T315I mutation, were achieved in an open-label phase II study evaluating the efficacy and tolerability of Blinatumomab in Ph-positive (Ph^+^) B-precursor ALL in the setting of relapsing or refractory to TKI-based therapy. This study findings helped the FDA expand its approval for Ph^+^ ALL indication in July 2017 (23). MRD is the most powerful prognosticator of relapse in ALL, and MRD-negative status has become increasingly clear that significantly associated with better event-free survival (24, 25). series of clinical trials have demonstrated the potency and efficacy of Blinatumomab in eradicating persistent or relapsed MRD in B-ALL patients with an increased MRD response rate (26–30). Based on these encouraging clinical findings, Blinatumomab acquired accelerated approval by the FDA to expand clinical indications to patients with MRD-positive ALL in 2018 (30). Blinatumomab has also been studied in phase 1/2 dose-escalation experiments for R/R Non-Hodgkin lymphoma (NHL), including diffuse large B-cell lymphoma (DLBCL), reaching an overall response rate of more than 40% (31, 32).

Although the clinical benefits for B-ALL patients from Blinatumomab is obvious, there are still problems with severe neurological events (encephalopathy, aphasia, and seizures) and cytokine release syndrome. In most studies, about 10% of patients reported ≥ grade 3 CRS and/or neurological complications. Dexamethasone or treatment interruption can alleviate these unfavorable side effects. In spite of this, about 10% of patients discontinued with Blinatumomab application because of treatment-related toxicity. Lunsumio (Mosunetuzumab) was recently granted conditional marketing authorization by the European Commission, a CD20 × CD3 T-cell binding bispecific antibody, for treating adult patients with relapsed or refractory follicular lymphoma (FL) who have received at least two prior systemic treatments. Roche is also working on Glofitamab, a CD20 × CD3 bispecific antibody with a different structure than Mosunetuzumab. Mosunetuzumab is similar to a natural human antibody but contains two different Fab regions, one of which targets CD20 and the other targets CD3. Glofitamab has a novel “2:1” structural pattern with two Fab regions targeting CD20 and one Fab region targeting CD3. This novel structural design allows higher binding to CD20 at the B cell surface.

The classification of hematological tumors is complex and varied. Acute myeloid leukemia (AML) is a genetically diverse disease defined by leukemic cell clonal proliferation. BiTEs are an effective treatment for AML because AML cells are especially vulnerable to the cytotoxic effects of functioning immune cells. CD33 expression is limited in non-hematopoietic tissues but is highly expressed in AML cells. The differential expression of CD33 on the surface of malignant AML cells makes it an ideal target for immunotherapy. AMG 330, the first CD33 × CD3 BiTE applied for acute myeloid leukemia (AML) patients, has shown promising cytolytic activity against AML cells in preclinical studies even at low CD33 antigen densities on target cells, making it a candidate for targeting a broad range of CD33^+^ leukemias (33–35). AMG330, similar to Blinatumomab, requires a 2-4 week cycle of continuous intravenous (IV) infusion. AMG330 was found to upregulate PD-L1 on primary ALL cells *in vitro*. As a result, when paired with PD-1/PD-L1 blocking therapy, AMG 330-mediated tumor cell lysis was dramatically increased (36, 37).

A bifunctional PD-1 × CD3 × CD33 immune checkpoint inhibitory T-cell engaging (CiTE) antibody simultaneously targeting PD-1, CD3 and CD33 has shown high therapeutic effect with complete AML (Acute Myelocytic Leukemia) eradication in preclinical experiments (38). Many clinical trials are ongoing with combination therapy of bispecific T cell-engaging antibodies and PD-1/PD-L1 axis inhibitors. The administration period depends largely on the structure of the antibody. The design of bispecific antibody-like Blinatumomab needs to consider the half-life. AMG673 will have an increased half-life of about 21 days in humans after fusing the binding domain of CD33 and CD3 to the N-terminal end of the IgG Fc region. In this way, the cycle time for intravenous infusion can be reduced. However, more attention needs to be paid to adverse effects. The drug AMV564 has a higher affinity for both antigens and possesses a tetravalent anti-CD33 × anti-CD3 tandem diabody (TandAb) structure with two CD3 binding sites and two CD33 binding sites. Whether T cells are overactivated is a concern for AMV564. Compared to AMG330, AMV564 is administered by continuous intravenous infusion at 14-day intervals. Preclinical studies *in vitro* and *in vivo* have demonstrated the ability of AMV564 to induce effective cytotoxicity to CD33^+^ AML cell lines in a dose-dependent manner.

CRS is the main toxic reaction in patients treated with CD33-targeted bispecific antibodies. Still, differences in the frequency and severity of CRS may depend on the leukemic load, the effector target ratio at baseline, the specific bispecific antibody structure, and its affinity for CD3.

More bispecific antibody for AML is undergoing clinical trials, such as CD123 × CD3 DuoBody (NCT02715011) and CD123 × CD3 DART (NCT02152956), and preclinical evaluation for adult patients, such as targeting CLL-1 and CD47. Target selection and efficacy assessment must be more cautious when treating AML in pediatric patients. More than 30% of AML pediatric patients have a highly tumor-specific target called MSLN. Recently, BsAbs targeting the MSLN and CD3 proximal area epitopes have increased lifetimes by increasing T cell activation and decreasing the tumor’s bone marrow AML cell load in MSLN-positive mice (39).

#### 3.1.2 BiTEs in solid malignancies

Bispecific T cell engagers (BiTEs) have revolutionized success in hematological malignancies treatment and revitalized the field of solid tumor immunotherapy with promising outcomes from preclinical and clinical trials. Even if the checkpoint inhibitors hold the majority of approvals in recent years in various solid tumor types, the T cell redirection and recruitment approaches are extremely promising. Contrarily, the solid tumor microenvironment has incredibly complex features that affect the infiltration, activity, and persistence of immune effector cells vital to anti-tumor immunotherapy (3, 11) (**Figure 1A**).

Catumaxomab (Removab) was the first bispecific T cell engagers (TCE) approved by the European medicines agency (EMA) for malignant ascites clinical intraperitoneal treatment in 2009 (40, 41). It is the intact trifunctional mouse/rat chimeric bispecific IgG antibody, with one arm from mouse IgG2a half-antibody identifying epithelial cell adhesion molecule (anti-EpCAM) on tumoral cells and another arm from rat IgG2b targeting CD3 subunit (anti-CD3) on T-cells. Additionally, the functional Fc fragment binds to different immune accessory cells with Fcγ receptors (FcγR), such as natural killer (NK) cells, dendritic cells (DC), monocytes, and macrophages resulting in T-cell-mediated lysis, ADCC, and accessory cells mediating phagocytosis (42). It employs humoral immunity, on the one hand, activates cellular immunity on the other, and delivers co-activation signals *via* attachable immune cells to eliminate truculent tumor cells, as well as allowing the body’s immune system to generate a specific immune memory, which acts as a cancer vaccine and inhibits tumor metastasis and recurrence. Catumaxomab’s effectiveness has been proven in key phase I/III research and other phase I/II studies (40).

However, intravenous applications of Catumaxomab were connected with severe adverse events like cytokine release syndrome (CRS) and dose-dependent liver toxicity (43), attributing to the off-target activity of other immune cells with FcγRs expressing, and it was withdrawn in 2017 from the market for some commercial reasons (44). The efficacy, safety, and tolerability of Catumaxomab are currently being studied in clinical trials for various indications involving patients with non-muscle invasive bladder cancer (NMIBC) and advanced or recurrent gastric carcinoma with peritoneal metastasis (NCT04819399; NCT04222114) (45).

Driven by the clinical success of Blinatumomab, various T cell-engaging BsAbs targeting solid tumors have been explored and evaluated in preclinical mouse xenograft tumor models and clinical trials. Various antigen being investigated for CD3 TCE bsAbs such as CEA, HER2, prostate-specific membrane antigen (PSMA), GlycoproteinA33 (gpA33), and Glypican 3 (GPC3), etc. (46–49). On-target off-tumor toxicity, a restricted number of effector cells in the tumor microenvironment (TME), and decreased T cell activation in tumors are all problems with CD3 bispecific antibodies in solid tumors. Anti-bispecific antibody designs and techniques for numerous challenges are also available. As a result, several novel formats of TCEs and prodrugs have been investigated for improved efficacy, such as a TCE with a monovalent CD3 binding region and a multivalent TAA binding region, which has been shown to effectively transform a poorly infiltrated tumor microenvironment (TME) into a highly inflamed TME with increased infiltration frequency of activated T cells. Besides, these new multivalent TAA binding regions enable avoiding on-target/off-tumor toxicities after being altered into low-affinity TAA binding domains since most TAAs are also generally expressed at low levels in normal tissue cells (50, 51).

Dionysos Slaga and colleagues have demonstrated a proof of concept. They have generated an anti-HER2/CD3 TCE BsAbs which can target HER2-overexpressing tumor tissue cells with selectivity and high potency while very low binding to normal tissue cells with low amounts of HER2 expressing, thus circumvents the risk of adverse effects to a certain extent (52). Current prodrugs utilize the characteristics of tumor microenvironment such as lower pH, oxygen levels, and proteolytic enzyme levels, allowing for tumor-specific activation of BsAb that are inactive in the circulation or normal tissue thus avoiding attack normal cells. Furthermore, the most fundamental strategy to avoid on-target/off-tumor toxicity is to choose targets only expressed in solid tumors. Immune-mobilizing monoclonal TCRs against cancer (ImmTAC) is a remarkable TCE bsAb format that targets MHC-presented intracellular neoantigen peptides on the surface of tumoral cells (53). This engineered antibody format consisted of an anti-CD3 scFv and TCR peptides with enhanced affinity, enabling to recruit and selectively activate a majority of polyclonal effector T cells to infiltrate to reverse the “cold” TME into an inflammatory TME and lysis cancer cells with low surface oncoprotein epitope densities in the context of HLA-A*0201 subsequently, which demonstrates significant anti-tumor efficacy (54). Tebentafusp (IMCgp100), an ImmTAC molecule, for example, targets melanocyte differentiation antigen polypeptide glycoprotein100 (gp100), showing clinical activity in Metastatic Uveal Melanoma with low tumor mutational burden. Tebentafusp outperformed a single-agent treatment of ipilimumab, pembrolizumab, or dacarbazine in an open-label phase 3 trial for extending overall survival in newly diagnosed patients with metastatic uveal melanoma (NCT03070392) (55).

Whether changing the valence of antibodies, bispecific antibodies in the form of prodrugs, or finding new targets can only solve the problem of on-target off-tumor, T cell infiltration and activity in solid tumors can mostly only be changed by the mode of administration or combination therapy. TAA-based targeting may underestimate the use of CD3 bispecific antibodies in solid tumors. A bispecific antibody against both PD-L1 and CD3 successfully connected T cells to PD-L1-expressing tumor cells, improved T cell cytotoxicity against multiple NSCLC-derived cell lines by releasing granzyme B and cytokines, and decreased tumor growth in mice (56). However, a more potent mechanism of action may exist for the PD-L1 × CD3 bispecific antibody, which was found to target dendritic cells rather than tumor cells in multiple homozygous tumor mouse models. Bispecific antibodies redirecting T cells to APCs by enhancing B7/CD28 co-stimulation to activate T cells may represent a general means of T-cell rejuvenation for durable cancer immunotherapy. PD-L1 × CD3 treatment is undoubtedly dual-acting by simultaneously blocking negative signaling (PD-L1) and engaging positive signaling (CD3). More targeting approaches are now conceivable with the identification of immune checkpoints, and more mechanisms of action are being examined (57).

Any single technique for treating solid tumors will either limit tumor growth or temporarily remove the tumor. A combination of multiple techniques is required to achieve the optimal treatment result. More target combinations and antibody screening modalities are being developed for solid tumors. Using Patient-derived organoids (PDOs), bispecific antibodies can be screened on a large scale, and their efficacy can be evaluated more reliably than in cell experiments.

### 3.2 BsAbs recruiting Natural Killer (NK) cells for tumor redirection

NK cells, derived from multipotent hematopoietic stem cells, were identified in 1975 and have been considered the first line of defense against tumor cells with the robust anti-tumor ability (58, 59). With MHC-independent cytotoxicity, cytokine synthesis, and immunological memory, NK cells have a unique anti-tumor function, making them crucial participants in the innate and adaptive immune response system. These cells are conventionally divided into two subtypes, CD56^dim^ CD16^+^ NK, and CD56^bright^ CD16^-^ NK cells. The former possesses powerful cytotoxicity and constitutes most of the peripheral blood and spleen subpopulation. At the same time, the latter is mainly equipped with immunomodulatory characteristics and constitutes a major subtype in lymph node tissues with weak cytotoxicity and maturity.

Diverse inhibitory and activating receptors are expressed on the surface of NK cells, determining the outcome of NK-cell activation by mediating the balance between those signals, which is pivotal for distinguishing and eliminating aberrant from normal cells through cytotoxic granules secretion based on TRAIL receptors and FAS ligand (FasL) expression as well as the release of other cytokines, growth factors, and chemokines. Inhibitory receptors on the surface of NK cells can recognize and bind to MHC Class I (MHC-I) molecules to alleviate autoimmune reactions. In contrast, the down-regulated expression of the MHC-I molecule can induce NK cell-mediated killing under cellular stress conditions, known as “missing self-recognition”. During tumorigenesis, the expression of MHC-I molecules is generally lost or in a defective condition for escaping from immune surveillance. Still, the unique characteristic of NK cells plays a necessary role in bypassing downregulated presentation of tumor neoantigens and effectively eliminating early aberrant cells (58).

A high frequency of NK-cell infiltration is usually connected with a better prognosis. However, Clara Degos and colleagues found an impoverished NK cell infiltration in the tumor microenvironment. IFN-γ secretion and cytotoxicity of Tumor-infiltrated NK cells are impaired, with significantly attenuated tumor-killing ability (60). Bispecific killer cell engager (BiKE) is a promising strategy to engage NK cells to tumor cells; Fc receptor (FcγRIII, CD16A)-mediated recruitment as a function of bsAb can be achieved by binding of CD16 on the surface of NK cells to the Fc region of bsAb, or by one end of a bi-specific antibody targeting CD16A (CD16A antibody). By inducing ADCC, the activating NK cell receptor CD16A (FcγRIIIA), which is mostly expressed on mature NK cells, might facilitate the destruction of tumor cells (61).

Both the composition and form of the BiKE affect the effectiveness of ADCC induction. The design of BiKE for Fc-mediated NK cell recruitment faces the challenge that the chosen form needs to ensure the effective binding of the Fc structural domain to CD16. Same as the Fc-mediated ADCC antibodies, the ADCC efficiency of BiKE, which recruits NK cells through partial regional CD16 antibody, is also similarly dependent on the choice of antibody’s form. ADCC-induced binding of the tumor-associated antigen (TAA) CD30 and CD16A is superior to monovalent CD30/CD16A binding in the dual CD16A-bound TandAb (tandem diabody) form than in the diabody form (62).

The AFM13 (ROCK^®^), a tetravalent bispecific anti-CD30 × anti-CD16A TandAb targeting CD30^+^ malignancies like Hodgkin lymphoma, has shown efficacy and cytotoxicity in an early clinical trial (NCT01221571) (63). Besides, in a phase 1b study aiming to evaluate the curative effect of AFM13 in combination with pembrolizumab to investigate further a rational treatment modality in patients with relapsed/refractory Hodgkin lymphoma (R/R HL), the objective response rate reached 88% at the highest treatment dose. The overall response rate is 83% for recipients with an acceptable safety profile and tolerability (64). Furthermore, AFM13 has reached a phase II clinical study to improve therapeutic efficacy by optimizing the dosing schedule (63). The AFM24, a different CD16A-based IgG1-scFv fusion BsAb that targets EGFR-expressing tumor cells with varying EGFR expression levels and KRAS/BRAF mutational status, has also demonstrated a strong potential for therapeutic application investigation and is now being studied in clinical trials (NCT04259450) (65).

Apart from targeting CD16A, a small number of studies have targeted activation receptors on NK cells, such as NKG2D, NKp30, and NKp46. A study constructed a homodimeric recombinant antibody combining two NKG2D-binding and two ErbB2 (HER2)-specific single-chain fragment variable (scFv) domains, linked by an IgG4 Fc region in a single tetravalent molecule, known as NKAB-ErbB2 (66).

The NKAB-ErbB2 increased lysis of ErbB2-positive breast carcinoma cells by peripheral blood-derived NK cells endogenously expressing NKG2D. NKG2D is unlike the other targets because mAb-mediated cross-linking does not result in cytokine release. In contrast, stimulation with soluble recombinant NKG2D ligands (MICA, ULBP-1, or ULBP-2) induces the expression of IFN-γ, GM-CSF and MIP-1β. A novel dual-targeting antibody composed of antibody cG7 and MICA was named cG7-MICA. The cG7 part is a natural antibody targeting CD24, and MICA is attached to the antibody behind CH3 *via* a G_4_S linker. When cG7-MICA coupled to CD24 on tumor cells, inducing NK cell-mediated cytotoxicity, HCC cells were identified by NK cells *via* MICA. As the Fc binds to its receptors on the surface of NK cells and macrophages, ADCC, CDC effects, and a longer half-life following engagement with neonatal receptors are all triggered (67). But shedding or downregulation of NKG2D ligands (NKG2DL) can prevent NKG2D activation, resulting in the escape of cancer cells from NKG2D-dependent immune surveillance.

There has been particularly little research into bispecific antibodies targeting NKp30 and NKp46 (68). CTX8573 is the first NKp30 × BCMA bispecific antibody that targets BCMA^+^ plasma cells and NK cells (69). The C-terminus of the antibody is attached to the anti-NKp30 Fab, and the mismatch is resolved using the same light chain. The Fc-terminus is given a de-fucose treatment to increase the impact on NK cells further. Intrinsic cells are effectively attracted to and activated by the binding of NKp30 and CD16A. Compared to monoclonal antibodies targeting CD16A, the ADCC potency is increased by more than 100 times using the NKp30 bispecific platform, which also maintains activity when CD16A is downregulated. Bispecific antibodies against NK redirection are still mainly used to treat hematological tumors. It is also unclear whether NK cells will enter solid tumors more readily than T cells for solid tumors. NK cells can produce relevant cytokines to attract other immune cells, which may further enhance the anti-tumor response. There is no doubt that strategies to increase the involvement of NK cells in anti-tumor response will be the future of tumor immunotherapy.

### 3.3 Bispecific antibody targeting immune checkpoint and co-stimulator for immune cell restoration

#### 3.3.1 Immune checkpoint

Clinical cancer therapy approaches have undergone a revolutionary change due to recent discoveries on the roles of immune checkpoints in allowing cancers to avoid the innate/adapted immune system (70, 71). Immune checkpoint receptors of co-inhibitory molecules such as programmed cell death 1 (PD-1) and/or cytotoxic T-lymphocyte-associated antigen 4 (CTLA-4) are critical in maintaining self-tolerance and avoiding immune-mediated adverse effects on the host. However, numerous studies have shown that the (TILs) exhibit substantially elevated co-inhibitory receptors, which represent an exhausted phenotype and limited anti-tumor action (72). In addition, preclinical evidence suggests that T cell response has been inhabited on account of the upregulated expression level of programmed cell death 1 ligand 1 (PD-L1) on the surface of malignant cells to conducive tumor cell’s immune escape and limit the efficacy of anti-tumor immunotherapies (73). Immune checkpoint blockades (ICBs) have been established based on the mechanism mentioned above to break those negative regulators that prevent pre-existing anti-tumor immune responses from being activated. Some ICBs have shown notable efficacy in various cancers and have entered routine clinical implementation (74). Besides, A clinical trial reported the 5-year outcomes that nivolumab (anti-PD-1) combined with ipilimumab (anti-CTLA-4) among advanced melanoma patients has resulted in sustained long-term progression-free and overall survival (52%) compared with nivolumab group (44%) and ipilimumab group (26%) (75).

The therapeutic idea of blocking two inhibitory immune checkpoints has led to the rational design and development of bispecific antibodies that simultaneously target two inhibitory checkpoints expressed on the surface of the same or different cells. This has been made possible by the innovative success of ICBs immunotherapies and the improved clinical benefit rate observed in patients who have received combined treatment with ICBs. MGD019 is a monovalent investigational PD-1 × CTLA-4 bispecific DART compound designed to increase CTLA-4 checkpoint blockage in the TME based on a PD-1 binding mechanism. This single-molecule showed complete blockade of the PD-1/PD-L1 axis and Variable Inhibition of CTLA-4 *in vitro* and is well tolerated in non-human primates with increased T cell proliferation and expansion. Furthermore, the first-in-human study with MGD019 is ongoing in patients with multiple advanced solid tumors. After the dose-escalation phase, the analysis revealed acceptable safety and objective responses in various tumor types typically unresponsive to checkpoint inhibitor therapy (NCT03761017) (76). MEDI5752, fusing an anti–PD-1 mAb and the variable binding domains of Tremelimumab (anti-CTLA4) onto a DuetMab backbone, optimally designed with triple amino acid mutations of the human IgG1 constant heavy chain to reduce Fc-mediated immune effector functions. Dovedi, S.J. et al. discovered that this engineered molecule preferentially localizes and inhibits CTLA4 on PD-1^+^ T cells and rapidly induces internalization and degradation of PD-1.

As a result, the affinity for the CTLA4 receptor is markedly increased and saturated, increasing clinical benefit and minimizing further harm. In addition, current first-in-human research using MEDI5752 to treat advanced solid cancers showed promising partial responses with acceptable side effects (77). Different from MEDI5752, AK104 is an anti-PD-1/CTLA-4 bispecific antibody with the symmetrical structure of 4-valent IgG1-scfv developed by Akeso Biology. AK104 can rapidly mediate independent endocytosis of PD-1 or CTLA-4 based on good antigenic differentiated binding with high retention in tumor tissue. Recently, Cadonilimab (AK104) has been approved in China for treating patients with recurrent or metastatic cervical cancer who have failed prior platinum-containing chemotherapy.

Nowadays, most of these BsAbs target the next wave of inhibitory receptors expressed on TILs with one binding arm and block the PD-1/PD-L1 axis with the other binding arm to reverse acquired T cell exhaustion-driven resistance. Dual immunomodulator MGD013 targets LAG-3 and PD-1. Both target molecules are expressed on T cells following antigen stimulation. Based on the DART^®^ form, MGD013 has been shown to effectively inhibit the binding of PD-1 to PD-L1 and PD-L2 while inhibiting the binding of LAG-3 and MHC II, which activate T cells by acting together. This bispecific antibody is in clinical phase I studies (NCT03219268). Similar preclinical dual immunomodulators include FS118 targeting PD-L1/LAG-3 and LY3415244 targeting PD-L1/TIM-3 (78, 79).

#### 3.3.2 Co-stimulatory molecules

Co-stimulation assists the immune system in determining whether responses to antigenic stimuli and co-stimulatory receptors have been utilized for cancer immunotherapy. The targets of immune co-stimulation mainly focus on the B7-CD28 and TNFR family. In the B7-CD28 family, CD28 and ICOS are the main co-stimulatory receptors. OX40, CD40, CD27, 4-1BB, GITR, and CD30 belong to the TNFR family. CD28, one of the first identified co-stimulatory molecules constructively expressed on the surface of T cells, contributes to lowering the threshold, which is critical for TCR-mediated T cell activation and subsequently results in enhanced T cell proliferation, cytokine production, and release, as well as cell survival (80). Six healthy volunteers who participated in the first human clinical trial for TeGenero’s CD28 hyperagonist antibody TGN1412 had severe cytokine release syndrome and multiple organ failure. Because antibodies activate T cells even when the TCR is not involved, they create an immunological response that targets everyone.

Other co-stimulatory receptors are promising targets, transiently expressed on activated T cells *via* TCR-mediated signal identification rather than constituent expression like CD28. The affinity of agonistic antibodies for their targets must be optimized, not maximized. It is not the affinity but the intermittent exposure of co-stimulatory receptors that may become more crucial. Excitatory antibodies, though, are still in their infancy compared to immune checkpoint inhibitors. Utilizing excitatory antibodies alone might not be the best course of action. Co-stimulatory receptors play a unique role, and PD-1 therapeutic effectiveness depends on the CD28/B7 co-stimulatory pathway. PD-1 is widely believed to inhibit signal transduction through T-cell receptors (TCR). The study indicated that TCR co-stimulatory receptor CD28 is the primary target of PD-1 signal transduction (81). Lung cancer patients who responded to PD-1 therapy had more CD28^+^T cells, suggesting that CD28 may predict treatment response. Toxicity can be largely avoided by combining CD28 antibodies with another target.

TSA × CD28 bispecific antibodies have shown little or no toxicity in humanized immune system mice or primate models when used alone or combined with PD-1 antibodies (82). As a result, it might offer a safe “off-the-shelf” combination immunotherapy that could greatly improve anti-tumor effectiveness and trigger long-lasting anti-tumor immunity. Preclinical research on CD28 triple antibodies has recently demonstrated considerable promise, and this therapy is now widely recognized in theory. Sanofi created an anti-HER2, anti-CD3, and anti-CD28 tri-specific antibody to target, stimulate, and prolong the lifespan of T cells in malignancies (83).

The dissociation constants for HER2, CD28, and CD3 are 1.28, 1.0, and 1.43 nM, respectively. Each antigen arm shows a comparable affinity to the analogous single antigen arm in the presence of the other two antigens, demonstrating minimal interference between the various arms. The tri-specific antibody could promote tumor regression at low doses and achieve effective tumor suppression in both high and low HER2 expressing tumors. It was also found that CD4 cells, but not CD8 cells, were critical in promoting tumor growth arrest. The CD137 (4-1BB) is the most promising target in studies targeting co-stimulatory receptors. To reduce the toxicity of systemic CD137 agonists to the liver while maintaining efficacy, targeting CD137 with a bispecific molecule that binds to the tumor-associated antigen (TAA) and confining CD137^+^ T cell agonists to the tumor microenvironment appears to be an ideal approach. Targeting non-tumor toxic cytokine release syndrome is decreased by triggering only antigen-exposed T cells (CD137^+^ T cells). Independent of MHC, CD137 activation can expand tumor-reactive memory T cells. Additionally, CD137-targeted antibodies may be more resilient to antigen loss than CD3-targeted antibodies. Nevertheless, the clinical development of bispecific antibodies has been severely hampered by dose-dependent hepatotoxicity found in clinical studies with co-stimulatory molecules recognizing agonistic antibodies (84–90).

Thus tumor-localized co-stimulatory bispecific antibodies have been developed to alleviate systemic toxicity after systemic effector T cell co-stimulation. PRS-343, engaging 4-1BB-specific Anticalin proteins to a modified variant of trastuzumab with a mutation modified IgG4 isotype to avoid the risk of ADCC and non–tumor-target activation of 4-1BB-positive lymphocytes, facilitates HER2+ tumor-localized co-stimulation of T cells with reduced peripheral toxicity (84). In addition, PRS-344/S095012, a synthesized tetravalent PD-L1/4-1BB bispecific antibody, showed stronger antitumoral activity and synergistic impact compared to the combination of mAbs *via* a tumor-localized 4-1BB-mediated activation (91). Preclinical models reflect that PRS-344/S095012-mediated 4-1BB activation depends on PD-L1, reducing the risk of peripheral toxicity and that 4-1BB co-stimulation occurs only in synchrony with TCR signaling, limiting its activity to antigen-specific T cells. Furthermore, DuoBody-PD-L1×4-1BB (GEN1046), the first-in-class bispecific immunotherapy agent, formed by the K409R and F405L mutations in the Fc CH3 region of two IgG1 antibodies and demonstrated pharmacodynamic immune effects and a manageable safety profile in a phase I trial of dose escalation in heavily pre-treated patients with multiple advanced refractory solid tumors (NCT03917381) (92).

In addition to bispecific antibodies, research for the CD28 and the 4-1BB target has been extended to triple and quadruple antibodies. However, the market for numerous distinct cancer antibody therapeutics is still in its infancy, and much research on its effectiveness and safety is still needed. Hepatotoxicity is being studied in the next generation of co-stimulation-targeted bispecific antibodies without compromising efficacy.

## 4 Bispecific antibody targeting non-immune cells in the TME for restricting tumor diffusion

**Figure 2:**
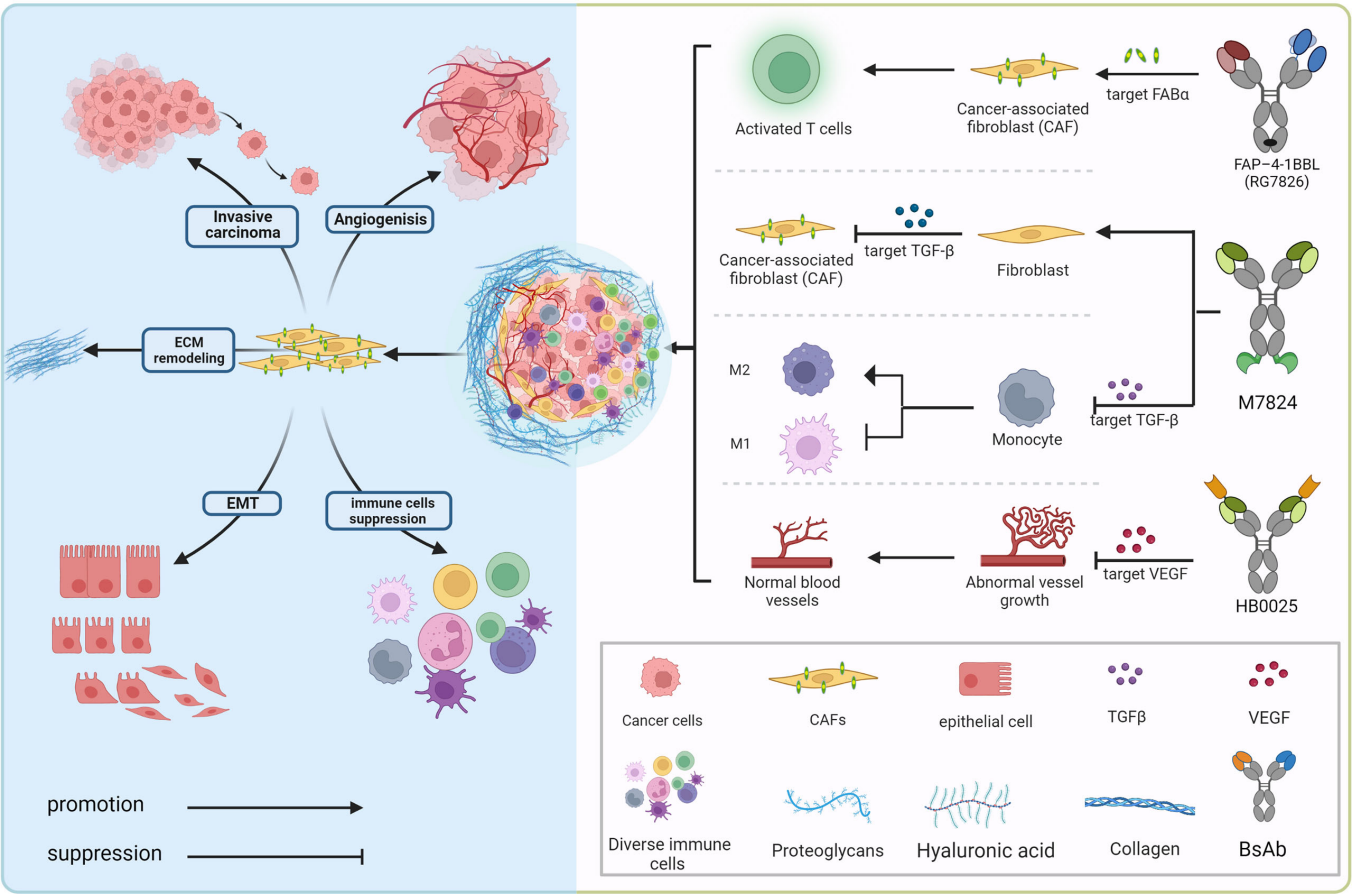
Bispecific antibodies exert anti-tumor effects in the immunosuppressive tumor microenvironment. In the complex tumor microenvironment, activated fibroblasts communicate with tumor cells, various inflammatory cells as well as stroma cells *via* secreting growth factors (TGFβ, VEGF, etc.) and other chemokines to provide potentially oncogenic signals and interact with the microvasculature, which induces an accelerated oncogenic extracellular-matrix microenvironment. BsAbs, aiming at blocking the interacting mechanism, transform the “cold” immune environment into the “hot” immune environment.

The tumor microenvironment mainly consists of tumor cells and their surrounding immune and inflammatory cells, cancer-associated fibroblasts (CAFs), nearby mesenchymal tissue, microvasculature, and various cytokines and chemokines (**Figure 2**). It can be roughly divided into immune microenvironment based on immune cells and non-immune microenvironment. Angiogenesis, the process by which new blood vessels emerge from an already-existing vascular network, is crucial to tumor growth, progression, and metastasis (93). During the process, low oxygen tension (hypoxia) is a significantly important component of the TME driving tumor angiogenesis, which could upregulate multifarious proangiogenic growth factors like VEGF, placenta growth factor (PlGF), and angiopoietin 2 (Ang2) that correlate with the formation of new vessels through directly engaging in vessel growth (94, 95). VEGF family (VEGF-F) and Ang1-2/Tie-2 pathway are equally important in mediating tumor angiogenesis, and Ang-2 regulates vessel maturation in the later stage of angiogenesis, which contributes to promoting vascular formation with VEGF in the different stages. Upregulated levels of VEGF and Ang-2 demonstrated a worse prognosis factor in various tumor types (96), the blocking of the signaling pathway Ang-2 shows the effect of tumor growth inhibition with the decreased vascular formation. It normalizes remaining blood vessels with increased pericyte coverage (97–99). Furthermore, the above blocking pathways have more significant effects when combined with anti-VEGFA drugs, even in certain tumor types with resistance (100–102).

In addition to showing strong anti-tumor, antiangiogenic, and micrometastasis growth reducing effects in subcutaneous and orthotopic syngeneic mouse xenotransplantations, Ang-2-VEGF CrossMab also exhibits these effects in patient or cell line-derived humanized tumor xenografts with acceptable side effects compared to Ang-1 inhibition combined with anti-VEGF treatment on physiologic vessel growth (103). Furthermore, Kloepper, Riedemann et al. found that dual Ang-2/VEGF (CrossMab, A2V) antibody can prolong the survival of mice bearing orthotopic syngeneic (Gl261) GBMs or human (MGG8) GBM xenografts based on only VEGF pathway blocking failing to enhance overall survival of patients with GBM (104). The logical combination therapy of immune checkpoints and angiogenesis provides greater therapeutic effects, according to Schmittnaegel et al. On the other hand, increased intratumoral immune effector cell activation results in increased PD-L1 expression in tumoral endothelial cells (105).

The PD-L1 blockade could prolong the angiostatic effects of angiogenic factors receptor inhibition, enhancing vascular normalization to a certain extent. However, lacking tumor-infiltrating lymphocytes is related to primary resistance to ICIs. At the same time, dysfunctional tumor vasculature restricts lymphocyte T cell permeating into tumors, thus limiting the curative effect of immune-checkpoint blockade. Therefore, rational dual therapy modalities of anti-angiogenesis and immune checkpoint blocking like PD-1/PD-L1 signal pathways have broad clinical applicability (91, 105). The HB0025, with dual recognition of VEGFR and PD-L1 based on mAb-Trap technology, has shown enhanced anti-tumor benefits than either single drug treatment (106).

In addition to VEGF, the Notch pathway’s essential ligand delta-like ligand 4 (DLL4) plays a key role in tumor neo-angiogenesis and regulates the VEGF pathway’s signaling to prevent excessive vascularization (107). Due to severe target toxicities (such as hepatotoxicity and pulmonary hypertension) seen in the clinic, the DLL4 monoclonal antibody’s development has been stopped. The research strategy then shifted to bispecific antibodies for VEGF and DLL4. Navicixizumab (OMP-305B83), an IgG2 humanized BsAb, targets DLL4 and VEGF simultaneously. The data from the phase 1a study showed manageable toxicities and anti-tumor activity in various tumor types, which encouraged an ongoing phase 1b clinical trial to further assess the curative effect in pre-treated ovarian cancer patients with platinum resistance (NCT03030287) (108). Besides, ABL001 (VEGF × DLL4), with enhanced biological anti-tumor activity in xenograft models than VEGF or DLL4 monoclonal therapeutic antibodies alone, is under phase 1 clinical study to evaluate combination therapy effect with heavy chemotherapy (NCT03292783) (92).

Cancer-associated fibroblasts (CAFs), ranking in the stromal cell population, which compose of diverse subpopulations with distinct functions in cancer, represent the most considerable component of the tumor microenvironment (TME) (109). Abundant studies have confirmed that CAF populations could exert different but mutual functions modulating tumor growth, proliferation, tumor metastatic dissemination, and extracellular matrix components remodeling, simultaneously correlated to immunosuppression TME establishment and chemoradiotherapy resistance (109–113). Recently, an in-depth study on the crucial role that CAFs play in the tumor immune microenvironment’s (TIME) pro-oncogenic functions has been done, showing CAFs as a promising therapeutic target (114). Fibroblast Activation Protein (FAP), a marker expressed on the surface of CAFs and detected in various cancer types of poor prognosis, has appeared as a novel strategy for targeted immunotherapies. Bispecific FAP-targeted 4-1BB ligand (RG7826), correctly assembled through CH1-CL domain crossover, knob into hole (KIH) amino acid mutation in the fragment crystallizable (Fc) domain, as well as mutations in CH1 (EE) and CL (RK) (12, 115), led to intensive IFN-γ and granzyme B secretion in human tumor samples while combined with tumor antigen-targeted (CEA) T cell bispecific (TCB) molecules (89, 116). A new FAP-DR5 (death receptor 5) tetravalent bispecific antibody called RG7386 aims to activate extrinsic DR5. In preclinical patient-derived xenograft models, hyperclustering dependent on the tumor cells’ apoptotic pathway and binding to FAP-positive stroma led to durable tumor reduction, which is currently being assessed in phase-I clinical research (117, 118).

## 5 Bispecific antibody changing TGF-β signal pathway to improve the tumor microenvironment

Transforming growth factor (TGF)-β is a multifunctional cytokine that plays a dual role, tumor suppressor or promoter, in a cellular or context-dependent manner, known as the TGF-β “paradox”. In early-stage tumors, the TGF-β pathway induces apoptosis and inhibits tumor cell proliferation. In contrast, it has a tumor-promoting role in advanced stages by regulating genomic instability, epithelial-mesenchymal transition (EMT), neoangiogenesis, immune evasion, and cell metastasis (**Figure 2**). Previous research suggests that response rates to TGF-β monoclonal antibody therapy are low, which could be related to the fact that it is not a tumor promoter. As a result, one of the primary avenues of advancement in this sector has been combination therapies, which include combinations with ICIs (e.g., PD-1/PD-L1 antibodies), cytotoxic drugs, radiotherapy, cancer vaccines, and so on.

The discovery that TGF-β antibody induces potent anti-tumor immunity when combined with PD-L1 antibodies was made in a study of patients with uroepithelial carcinoma who metastasized after receiving PD-L1 antibodies (119). In this study, CD8^+^ T cells were found in the patients’ tumor interstitium containing fibroblasts and collagen but not in the tumor interior, TGF-β signal limits T cell infiltration. At the same time, some researchers fused TGF-β receptor II and PD-L1 antibodies into a tetravalent BsAb, M7824, and found that the bispecific antibody had better anti-tumor effects than the monotherapies in homozygous mouse models of breast and colon carcinoma. The Phase I clinical study (NCT03917381) of M7824 patients with non-small cell lung cancer (NSCLC) evaluated its efficacy and safety. The median follow-up was 51.9 weeks, with an objective remission rate (ORR) of 21.3%, partial remission (PR) of 21.3%, stable disease (SD) of 16.3%, and disease progression (PD) of 48.8% for all patients; disease control rate (DCR) of 40%; overall survival (OS) of 13.6 months; median progression-free survival (PFS) of 2.6 months; The 12-month PFS rate was 20.1%; median duration of remission (DOR) was 14.1 months. A dose of 1200 mg was determined to be the recommended dose for the Phase II study. But German Merck and collaborator Glaxo finally announced that a Phase III (INTR@PID Lung 037) interim analysis of bintrafusp alfa (M7824) showed that it could not outperform PD-1 antibody Keytruda. Bintrafusp alfa was then stopped as a single-agent second-line treatment for locally progressed or metastatic biliary tract carcinoma (BTC) in Phase II INTR@PID BTC 047 due to failure to reach the primary endpoint. Bintrafusp alfa has failed four clinical trials in a row since 2021. M7824 is also being tested in various indications, including esophageal, biliary tract, and gastric cancers. With the discovery that transforming growth factor-β (TGF-β) inhibits T helper cell (Th2)-mediated cancer immunity, researchers constructed a bispecific antibody targeting CD4 and TGF-β, 4T-Trap, which selectively inhibits TGF-β signaling by CD4^+^ T cells in lymph nodes (120, 121), leading to cancer cell apoptosis and vascular rearrangement. Due to TGF-β multifunctionality and the potential for major side effects from the total blockade, inhibition of TGF-β in cancer therapy has not been successful. However, 4T-Trap focuses TGF-β blocking compounds directly on CD4^+^ T cells to minimize adverse effects. And combining 4T-Trap with a VEGF inhibitor may help to prevent the spread of vascular-mediated malignancy.

Due to the diversity of TGF-β signaling, combinations are likely to be effective only when TGF-β is the tumor-promoting signal. The efficacy of anti-TGF-β must be carefully analyzed when TGF-β exerts tumor-suppressive effects or when the receptor for TGF-β is mutated. In addition, the combination of drugs may cause a strong immune response in patients; whether the patient can tolerate it and the deepening of side effects is also a question worthy of consideration. A deeper understanding of the communication and interactions between the various components of the tumor patient’s organism and TGF-β signaling is key to improving clinical efficacy.

## 6 Approaches to avoid on-target off-tumor adverse effects

A reinvigorated anti-tumor immunity can be a double-edged weapon while many programs explore alternative modes of action correlated with bispecific antibodies’ target pathways. The efficacy of genetically modified bispecific antibodies against cancer has increased greatly at the expense of improved toxicities in normal tissues and systemic cytokine release immune responses (122, 123). Strategies of conditionally activating T cells within tumors and modifying target affinities to mitigate or conquer the “on-target off-tumor” adverse effect of bispecific antibodies have been taken into consideration and investigation (52, 124–126).

To avoid T cell autoreactivity to normal target-expressing tissues while there is generally lacking tumor-specific targets in solid tumors, Slaga, D., and colleagues have designed and exploited a T cell-dependent bispecific (TDB) antibody with a bivalent low-affinity HER2 recognition binding domain which can selectively target HER2-overexpressing tumor cells from normal human tissues with low amounts of HER2 expressing (52). Besides, antibody binding affinity is a major factor for overall tolerability, while the higher affinity for CD3 is related to rapidly increasing peripheral cytokine concentrations. While having no impact on anti-tumor effectiveness, anti-HER2/CD3 TDBs with lower CD3 binding affinity is better tolerated *in vivo.* Higher HER2 affinity aids in tumor-killing action but also causes more severe toxicity, including cytokine release syndrome, in HER2-expressing tissues. a dose-fractionation technique, which offers an application strategy for the affinities-modulated antibodies, has been used to address such a problem (124).

Disorganized tumor tissue growth and rapid cell division contribute to complicated extracellular features of the TME, such as a hypoxia environment with low pH, increased extracellular matrix remodeling, and upregulated proteolysis, which has contributed to the exploration of conditionally activating T cells in the TME based on the preferentially binding of BsAbs at hypoxic extracellular conditions as well as local liberation of the BsAbs antigen-binding sites released by tumor-associated proteases (126–135). Preclinical research has shown that T cell recruiting BsAbs (TCBs) conditionally activated by intratumorally proteolytic cleavage can prolong therapeutic windows, successfully avoid dose-limiting toxicities, and significantly prolong tumor regression (131–133). Furthermore, Geiger, M. and colleagues have generated a protease cleavable activated anti-folate receptor 1 TCB (Prot-FOLR1-TCB) *via* masking the anti-CD3 binding domain with an anti-idiotypic anti-CD3 scFv N-terminally connected to the anti-CD3 variable heavy chain through a protease cleavable linker, which has shown validly releasing of the anti-CD3 binding moiety in active proteases enriched tumor microenvironment, thus effectively reducing potential on-target toxicity through sparing normal tissues with low degrees FOLR1 expression while mediating efficient anti-tumor ability in FOLR1-positive tumor tissue (126). Besides, binding sites of the BsAb assembling intratumorally from two half-molecules is an ideal approach to further increasing tumor selectivity (125, 136).

## 7 Conclusion and prospect

As a result of the FDA’s approval of Blinatumomab for the treatment of recurrent ALL and the ongoing need for BsAbs, novel formats aimed at enhancing therapeutic efficacy and safety have been developing for the treatment of solid tumors as well as hematologic malignancies. BsAbs have gained momentum over the past decade. Despite promising progress in the clinical application field of bispecific immunomodulatory antibodies in part of human tumor types, more prominent anti-tumor efficacy in most solid tumors still needs constant exploration. Furthermore, dozens of BsAbs with different targets combinations have exhibited potent anti-tumor effect in preclinical studies, but most of the positive preclinical outcomes could not be further validated in the clinic. With increasingly diverse BsAbs entering preclinical and clinical trials, various challenges have emerged hampering the development of BsAbs. The development of BsAbs is dimensionally more difficult than that of a monoclonal antibody. Selecting the optimal targets combination is only the first step, followed by the right choosing of a rational format and designing the molecule according to the targets as well as the biology of the diseases. Besides, inappropriate clinical design and administration regimens will expose patients into significantly higher toxicities, which can be avoided *via* optimization of treatment strategies, dosage, timing, and sequence to some extent. We anticipate that more comprehensive exploration in the field of bispecific immunomodulatory antibodies will broaden the prospect of cancer immunotherapy.

## Author contributions

All authors listed have made a substantial, direct, and intellectual contribution to the work, and approved it for publication.

## Funding

This work was supported by 1.3.5 Project for Disciplines of Excellence, West China Hospital, Sichuan University (Grant No. ZYJC21043), the National Natural Science Foundation of China (31971390), and Sichuan Science and Technology Program (2021YFH0142).

## Conflict of interest

The authors declare that the research was conducted in the absence of any commercial or financial relationships that could be construed as a potential conflict of insterest.

## Publisher’s note

All claims expressed in this article are solely those of the authors and do not necessarily represent those of their affiliated organizations, or those of the publisher, the editors and the reviewers. Any product that may be evaluated in this article, or claim that may be made by its manufacturer, is not guaranteed or endorsed by the publisher.
